# Herbert Coddington Major (1850–1921)

**DOI:** 10.1007/s00415-023-12150-x

**Published:** 2023-12-20

**Authors:** Andrew J. Larner

**Affiliations:** Department of Brain Repair and Rehabilitation, Institute of Neurology, University College London, London, UK

**Keywords:** Comparative neurology, Herbert Major, Neuropathology, West Riding Asylum

## Abstract

Herbert Coddington Major (Fig. 1) was a late nineteenth century pioneer in neuropathology and comparative neurology. No previous biographical article has been identified, suggesting he is now almost totally, yet unjustifiably, forgotten.

Born in Jersey, the baptism record for Herbert Coddington Mauger [*sic*] in St Helier gives his date of birth as 30th January, whereas his marriage entry, as Major, at St. Brelade’s Church, Jersey, on 20th February 1906 says “(Born abt 1850)”. His obituary notice states that he died on 13th September 1921 [1]. Following early education in Jersey, he undertook his medical studies in Edinburgh where he attended Thomas Laycock’s class in Medical Psychology. Graduating in 1871, he later won a gold medal for his thesis (of 67 pages) entitled *Histology of the brain in apes* in 1875 (Fig. 1).

**Fig. 1:**
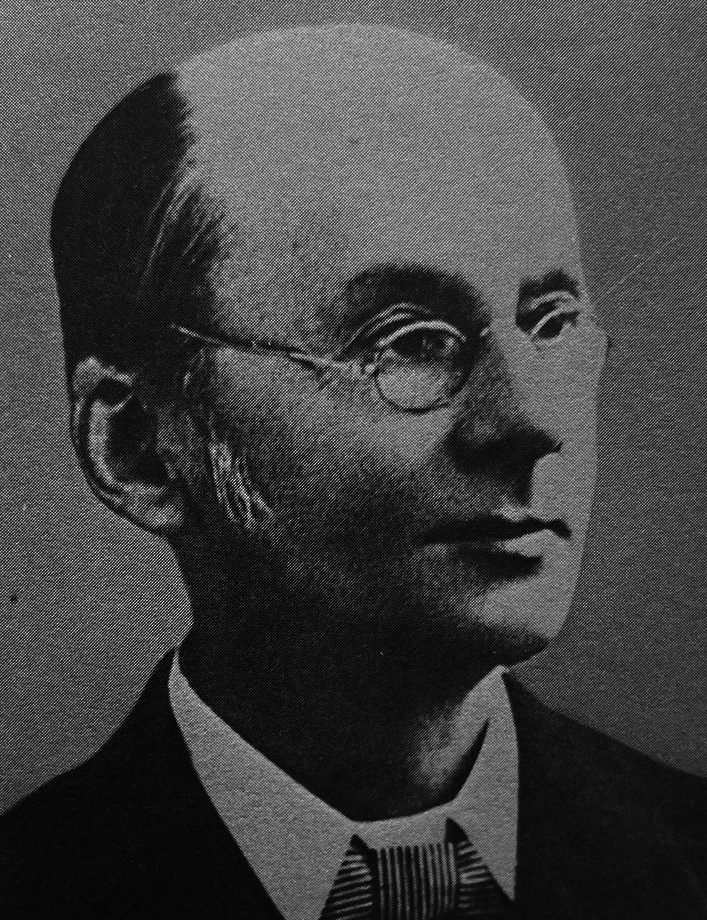
Herbert Coddington Major (1850–1921)

By this time Major was working at the West Riding Pauper Lunatic Asylum at Wakefield, Yorkshire, having been appointed Clinical Assistant (1871) and then Assistant Medical Officer (1872) by the asylum superintendent, James Crichton-Browne (1840–1938), who was then developing the institution into a significant neuroscientific research school [2]. From an early stage, Major took an interest in brain pathology, using the dedicated pathological laboratory facilities which Crichton-Browne had developed, this work facilitated by the fact that even junior Assistant Medical Officer posts were combined with that of Pathologist. In August 1872, at the Psychological Section of the British Medical Association annual meeting, Crichton-Browne showed “some beautifully prepared sections of Brain-Structure in Health and Disease, the work of Dr. Herbert C. Major, of the West Riding Asylum” (*BMJ* 1872;2:221). Major was an early advocate for staining procedures on brain tissue [3], at a time when most asylum post-mortem brain examinations were simply macroscopic.

Major was a significant contributor to the asylum house journal, the *West Riding Lunatic Asylum Medical Reports* (*WRLAMR*), published annually between 1871 and 1876. He authored six papers in all, published between 1872 and 1876, more than any other contributor save Crichton-Browne (Hughlings Jackson and David Ferrier were also contributors) [4]. These works ranged from single detailed case studies (“On the minute structure of the cortical substance of the brain, in a case of chronic brain wasting”), through case series (“Observations on the histology of the brain in the insane” and “Observations on the histology of the morbid brain”), to more technical papers (“A new method of determining the depth of the grey matter of the cerebral convolutions”). The latter paper used an instrument of his own design, the tephrylometer, to measure the thickness of grey matter.

Lest it be thought he spent all his timing gazing down a microscope, it is clear that Major did his share of clinical work at the asylum. He undertook most of the work on which Clifford Allbutt’s *WRLAMR* paper “The electric treatment of the insane” was based, and Crichton-Browne used Major’s clinical notes concerning a patient with puerperal mania, pelvic haematocele, and sudden death as the basis for a report in the *Lancet* (1874;1:54). Major was also present at some of the annual medical *conversazione* held at the asylum, presiding over stalls showing microscopic preparations from the asylum collection. He also gave lectures on Mental Diseases at the Leeds School of Medicine, a role initiated by Crichton-Browne.

As indicated by his thesis, Major’s interests extended beyond the human brain. He had a particular interest in “The histology of the island of Reil,” the subject of his final paper in *WRLAMR*, and performed a comparative study of this structure in apes [5]. John Hunter Arbuckle, a former Clinical Assistant at Wakefield Asylum, later described Major as “the first authority on the minute structure of the cerebral cortex of man and monkeys”. In addition to examining senile atrophy in the human brain, Major also examined ageing dog, horse, and cat brains in search of analogous changes, as reported in his penultimate *WRLAMR* paper “On the morbid histology of the brain in the lower animals”. Subsequent comparative studies included an examination of the brain of a Chacma baboon, obtained from the Zoological Gardens, London [6], and of a white (beluga) whale obtained from the Westminster Aquarium [7].

After Crichton-Browne’s resignation from the Wakefield asylum superintendency in late 1875, Major was appointed in his place in early 1876. It was a hard act to follow. Major co-edited the sixth and final volume of *WRLAMR*, its termination presaging the emergence of *Brain* in 1878 (to which Major did not contribute). Another aspect of Major’s “inheritance” was the probability that further asylum accommodation would be needed in the West Riding due to the apparent increase in lunacy. This may have stimulated his interest in statistical tables of the causes of insanity [8], a scheme which spread to asylums nationally in the following decade [9]. Unlike his predecessor Major opposed the provision of beer as an item of patients’ diet and so phased it out, a move unlikely to make him popular with some of the patients.

The administrative burdens of asylum superintendency seem to have weighed heavily on Major. Although he still published occasional case reports with detailed neuropathological correlation (e.g., [10]), these were fewer. He resigned the superintendency in 1884. As the *Journal of Mental Science* (1885;30:654) noted, “Dr. Major has … by his individual efforts in varied histological research advanced science *pari passu* with the fulfilment of the routine duties of administration, although unfortunately at the sacrifice of his health.” His successor as superintendent was William Bevan-Lewis (1847–1929), also a noted neuropathologist.

Major later resumed clinical work, as consulting physician at Bradford Infirmary, before moving to Bedford, then back to Jersey where he was married to Mary Ann Balleine in 1906. He had recently moved to Oxford at the time of his death [1]. Some 10 years later, the Medical Charitable Society for the West Riding of the County of York received a legacy of £500 under the terms of the will of the late Mrs. M.A. Major “in memory of her husband, Dr. Herbert Coddington Major, formerly of the West Riding Asylum” (*BMJ* 1931;2:270–271).
